# Locum doctor working and quality and safety: a qualitative study in English primary and secondary care

**DOI:** 10.1136/bmjqs-2023-016699

**Published:** 2024-04-16

**Authors:** Jane Ferguson, Gemma Stringer, Kieran Walshe, Thomas Allen, Christos Grigoroglou, Darren M Ashcroft, Evangelos Kontopantelis

**Affiliations:** 1 Health Services Management Centre, School of Social Policy, University of Birmingham, Birmingham, UK; 2 Alliance Manchester Business School, University of Manchester, Manchester, UK; 3 Manchester Centre for Health Economics, Division of Population Health, Health Services Research and Primary Care, University of Manchester, Manchester, UK; 4 Danish Centre for Health Economics, University of Southern Denmark, Odense, Denmark; 5 NIHR Greater Manchester Patient Safety Research Collaboration (PSRC), Division of Pharmacy and Optometry, Faculty of Biology Medicine and Health, University of Manchester, Manchester, UK; 6 Division of Informatics, Imaging and Data Sciences, University of Manchester, Manchester, UK; 7 NIHR School for Primary Care Research, Centre for Primary Care, Division of Population Health, Health Services Research and Primary Care, University of Manchester, Manchester, UK

**Keywords:** Governance, Health services research, Patient safety, Qualitative research, Quality improvement

## Abstract

**Background:**

The use of temporary doctors, known as locums, has been common practice for managing staffing shortages and maintaining service delivery internationally. However, there has been little empirical research on the implications of locum working for quality and safety. This study aimed to investigate the implications of locum working for quality and safety.

**Methods:**

Qualitative semi-structured interviews and focus groups were conducted with 130 participants, including locums, patients, permanently employed doctors, nurses and other healthcare professionals with governance and recruitment responsibilities for locums across primary and secondary healthcare organisations in the English NHS. Data were collected between March 2021 and April 2022. Data were analysed using reflexive thematic analysis and abductive analysis.

**Results:**

Participants described the implications of locum working for quality and safety across five themes: (1) ‘familiarity’ with an organisation and its patients and staff was essential to delivering safe care; (2) ‘balance and stability’ of services reliant on locums were seen as at risk of destabilisation and lacking leadership for quality improvement; (3) ‘discrimination and exclusion’ experienced by locums had negative implications for morale, retention and patient outcomes; (4) ‘defensive practice’ by locums as a result of perceptions of increased vulnerability and decreased support; (5) clinical governance arrangements, which often did not adequately cover locum doctors.

**Conclusion:**

Locum working and how locums were integrated into organisations posed some significant challenges and opportunities for patient safety and quality of care. Organisations should take stock of how they work with the locum workforce to improve not only quality and safety but also locum experience and retention.

**WHAT IS ALREADY KNOWN ON THIS TOPIC**

- Despite longstanding policy concerns about the implications of locum working for quality and safety, there has been little empirical research. Understanding how organisations engage, support and work with locums and how locum doctors integrate and interact with the complex and changing systems in which they work is essential if quality and safety are to be improved.

**WHAT THIS STUDY ADDS**

- This qualitative study examines the perspectives of locums, patients and people who work with locums to identify the implications of temporary medical working for quality and safety.

**HOW THIS STUDY MIGHT AFFECT RESEARCH, PRACTICE OR POLICY**

- Organisations should examine how they engage, support and work with locums. Organisations and locums need to reflect on whether their practices support a collective approach to patient safety and quality of care.

## Introduction

Temporary doctors, often known as locums, are a vital resource that enable healthcare organisations to deliver care by flexing capacity and covering staffing gaps. In the United Kingdom, all doctors, other than those in their first year of training after qualifying, can work as a locum. Locum work can vary from very short-term (a single shift) to longer-term assignments (weeks, months or even sometimes years). Locums find work through various platforms, including locum agencies, online job platforms, professional networks or word of mouth. Locum agencies typically have some governance responsibilities (such as compliance with regulations and licensing requirements), but the extent of these responsibilities varies and the NHS in England has no oversight over how recruitment agencies operate. Despite concerns among policymakers, healthcare providers, professional associations and professional regulators about the implications of locum working for quality and safety and cost,^1–3^ there is limited robust empirical research to evidence or support those concerns.

The workforce retention crisis is a significant challenge in healthcare internationally^4–6^ and persistent understaffing poses a serious risk to patient safety.^7 8^ In the UK, high doctor turnover has been linked to poorer service and health outcomes^9^ and has led NHS trusts and general practices (GPs) to be ‘overly reliant’^3^ on temporary staff to fill rota gaps.^10 11^ Expenditure on temporary staff in the NHS in England increased from £3.45 billion to £5.2 billion between 2021 and 2022.^3 12^ The NHS Long Term Workforce Plan aims to reduce reliance on temporary staff and make substantive employment the most cost-effective and attractive option.^3^ However, with the vacancy rate in the NHS projected to increase,^13^ locums are likely to continue to be essential to maintaining service provision, especially in shortage specialities such as psychiatry.^14^

An obvious implication of locum working is a reduced likelihood of organisational and team integration,^15^ familiarity and a shared understanding of ‘the way things are done around here’.^16^ Locums are likely to be less familiar with teams and other contextual factors relevant to providing safe and effective care^17^ and more likely to be situated on the periphery of organisational structures, teams and governance systems^1 18^ Teamwork represents a powerful process to improve patient care,^19 20^ and trust, shared understanding, communication and collaboration have been associated with better patient outcomes.^21 22^ The ability of healthcare teams to develop and maintain team situational awareness, or a shared perception, comprehension and subsequent projection of what is going on in complex and changing clinical environments, has been described as crucial for patient safety.^23 24^ Through participation and working together,^25^ teams gain an understanding of the roles, skills and competencies of others to demonstrate ‘collective competence’,^26 27^ which is critical for healthcare delivery,^28 29^ and existing research on locums suggests a need for better integration into teams to improve quality and safety.^30 31^

Context matters for patient safety and quality improvement,^32 33^ yet the limited evidence^17^ relating to locums practice is largely ‘acontextual’ and tends to ignore the role of the organisation in the integration of temporary staff, focusing instead on the potential risks locums present as individual clinicians,^17 30^ which is perhaps unsurprising given the liminal space locums occupy. In the UK, responsibility for the quality and safety of healthcare services is shared primarily between organisations and the individual professionals working within them.^34^ Organisations are responsible for creating systems and environments that promote and protect clinical governance and enable all doctors to meet their professional obligations, while doctors are expected to participate in the systems and processes put in place by regulators and organisations to protect and improve patient care.^35^ However, NHS trusts and primary care organisations procure the services of locum doctors without assuming the responsibilities normally associated with an employer–employee relationship^30^ and locums often struggle to participate in teams and governance systems that were designed for doctors working in conventional employment relationships.^18 36^

There is longstanding debate about the role of individual accountability in patient safety and how responsibility is distributed between organisations and individuals.^37^ A systems approach reasons that adverse events are likely to occur as a result of system failures rather than individual failures,^38^ and patients are protected from mistakes by well designed systems and environments that promote safety cultures.^39^ But locums are often positioned at the periphery of these systems,^30^ and doctors who are new to and also peripheral to organisations, and organisations who are inexperienced with and unsupportive of locums are unlikely to be able to perform optimally.^40^

The aim of this research was to provide evidence on how locum working arrangements impact quality and safety and the implications of locum working for patients, locums and health service organisations in primary and secondary care in the English NHS. Locum doctors are an essential and growing part of the healthcare workforce^1^ who have been largely ignored in healthcare workforce research. This research addresses a gap in the empirical evidence base on how locum doctor working arrangements affect quality and safety, and provides, for the first time, an in-depth exploration that includes perspectives from patients, locums and the people they work with.

## Methods

### Study design and setting

A qualitative semi-structured interview and focus group study was conducted with locums, people working with locums, and patients with experience of being treated by locums. Participants were purposively sampled through 11 organisations, including NHS trusts, primary care practices, statutory NHS bodies and locum agencies. Locum doctor participants were recruited through these organisations, locum recruitment agencies and networks. We used purposive, snowball and convenience sampling, drawing on intelligence from stakeholders, including our project advisory group, to identify and recruit organisations and participants. Patient participants were recruited through patient and contributor forums. The forum involved active partnership between patients and researchers in the research process to develop research which is relevant and useful to patient and public needs. Participant demographics were monitored to ensure representation across a broad range of roles in primary and secondary care and to increase diversity in terms of gender and ethnicity (see table 1).

**Table 1:** Characteristics of study participants

Characteristic	Professionals (n=88)	Patients (n=42)
Mean age (SD)	46 (11.0)*	59 (14.4)†
Gender (women)	49 (56)	24 (57)
Ethnicity		
White English	45 (51)‡	30 (71)†
Any other white background	12 (14)‡	4 (10)†
Mixed/multiple ethic group	3 (3)‡	1 (2)†
Asian/Asian British	15 (17)‡	2 (5)†
Any other Asian background	2 (2)‡	0†
Black/African/Caribbean/Black British	2 (2)‡	1 (2)†
Other ethnic group	1 (1)‡	0†

Data are presented as frequency (%) unless stated otherwise.

*n=81.

†n=38.

‡n=80.

### Data collection

Three semi-structured interview and focus group guides were developed for use with locums, people working with locums and patients with experience of being treated by locums (as shown in online supplemental files 1-3). Our previous review of the literature relating to quality and safety and locum work^17^ informed the schedules as well as the initial coding and thematic development. Schedules were also refined and informed by our patient and public involvement (PPI) forum and our project advisory group. Each schedule was intended to explore locum doctor working arrangements with a particular focus on understanding how locum doctor working may affect the safety and quality of care and what strategies or systems organisations and individuals used to assure or improve quality and safety. The topic guides for locums and people working with locums also covered governance and support, the impact of the COVID pandemic and policies and initiatives used to support locums.

10.1136/bmjqs-2023-016699.supp1 Supplementary data

10.1136/bmjqs-2023-016699.supp2 Supplementary data

10.1136/bmjqs-2023-016699.supp3 Supplementary data

### Analysis

Interviews and focus groups were transcribed verbatim by a professional transcription company and organised into codes and themes using the software package NVivo.^41^ Reflexive thematic analysis (RTA)^42^ was used and involved familiarisation with the data by reading and re-reading the transcripts and field notes; coding the dataset and collating all relevant data extracts; generating initial themes by examining the codes and collated data to identify significant broader patterns of meaning across the dataset; reviewing themes by questioning whether themes answered the research question and told a convincing story of the data and combining, splitting and discarding themes as necessary; defining and naming themes by developing a detailed analysis of each theme; and finally the analytical write up which positioned the analysis in relation to existing literature.^43^ RTA acknowledges the active role of researchers in knowledge production and the researcher’s subjectivity as the analytic resource.^42^ RTA recognises interpretive variability between researchers based on differences in their knowledge and skills, theoretical assumptions and differences in how they responded to the dataset is acknowledged and expected.^42^ The research team worked reflexively discussing their personal biases and their potential impact on the research at regular meetings throughout the data collection and analysis period. Our PPI forum were also involved in data collection and analysis, and offered a form of triangulation to enhance rigour, challenge and alternative interpretations of the findings.^44^ Analysis adopted a constructionist epistemology, in that while we acknowledged the importance of recurrence in generating themes, meaning and meaningfulness were the central criteria in the coding process.^42^

After themes were developed, an abductive approach was taken to position findings against a background of existing theory and knowledge.^17 30^ This provided a way of constructing empirically based theorisations without confining theory to predefined concepts.^45^ This approach integrated inductive data-driven coding with deductive theory-driven interpretation; aiming to find a middle ground between inductive and deductive methods and the most logical solution and useful explanation for phenomena.^45^

## Results

We conducted 130 interviews with 88 participants who worked in healthcare and 42 patients took part in focus groups and one-to-one interviews. Participants included locums, permanently employed doctors; nurses and other health professionals; medical directors/clinical leaders; responsible officers (ROs are accountable for local clinical governance processes and focus on the performance of doctors) and appraisers; leads for medical staffing and clinical governance and practice managers (see table 2). Three experienced qualitative researchers (JF, GS and KW) and two members of the PPI forum (MM and MS) carried out five focus groups with 30 patients, and JF and GS carried out 12 one-to-one interviews. Data were collected between March 2021 and April 2022 during the COVID pandemic using video conferencing software (n=126) or over the phone (n=4) at a time convenient to participants. Interviews and focus groups ranged in length from 23 to 171 min, with the average interview being 59 min.

**Table 2:** Healthcare organisations and participant roles

Healthcare organisation type	Participant roles	Total
Primary care	GP x 6 Managing director Non-clinical partner Nurse Practice manager x 5	14
Secondary care	Assistant psychologist Clinical director Clinical lead Clinical pharmacist Consultant x 9 Deputy medical director x 2 Director of patient experience Medical director x 2 Medical staffing x 6 Nurse x 3 Responsible officer x 3 Senior manager x 3	33
Primary care	Locum	19
Secondary care	Locum	12
Primary and secondary care	Locum	3
Locum agencies	Locum agency responsible officer x 4 Director	5
Other	Advisor to NHS x 2	2
Total		88

### Thematic framework

Our findings are presented under five broad and interrelated themes that examine how locum work relates to and impacts quality and safety: ‘familiarity’ with an organisation and its patients and staff; ‘balance and stability’ in services with lots of locums; ‘discrimination and exclusion’ towards locums and their effects; ‘defensive practice’ by locums; and the positioning of locums outside clinical governance arrangements.

### Familiarity: knowing who, where and how

Locums described often working in unfamiliar environments, sometimes with minimal induction and varying levels of support. Unfamiliarity, lack of access to or other restrictions on computer systems, policies, procedures and buildings meant that locums were not always able to do their job safely, productively or effectively.

That’s probably the biggest sort of safety aspect that sticks in my mind, is that it is unbelievably frustrating to have to learn a whole new set of patients from day to day … when I was signed up to four different hospitals, plus the locum agencies, I very quickly realised that not only is it the fact that you don’t know the patients from day to day, if you’re chopping and changing site the whole time, then store cupboards are laid out differently, ways of contacting relevant staff members are different, you’ve got to recognise what code to put in to bleep someone that’s different at every single site. (Interview 23, locum, secondary care)

Locum working sometimes created extra work for permanent staff who were responsible for inducting, training and supervising locums. The amount of additional workload was dependent on contextual factors, such as the experience of the locum, organisational support and length of placement, access to systems and what terms and conditions locums or organisations had negotiated. Locum reliance on permanent staff meant that care could be delayed, partially completed or not completed at all, which sometimes caused resentment.

Some of the things that we don’t … like, for example, procedures of limited clinical value that we don’t refer in for, they won’t know about those in our areas … So they’ll do referrals that we then will get pulled on. They’ll maybe prescribe medications that are not first line medications within our own formulary. So we see quite a bit of that, you know, there’s quite a lot of tidying up to be done afterwards or work. They generate that. So whilst we meet the patient numbers, they create a lot of work for the rest of the team. (Interview 3, practice manager, primary care)

Locums mitigated risks related to working in unfamiliar environments by avoiding organisations considered chaotic or unsafe, working below their grade to avoid having responsibility in unfamiliar organisations where they may not be supported or included in the team or working in a limited number of organisations to increase familiarity.

Most locums take jobs, locum work below their grade. So a person who’s at a registrar level would take a locum work as an SHO (senior house officer), because they don't know the trust that well. (Interview 55, locum, secondary care)

However, lack of familiarity and discontinuity could at times be beneficial for patients and organisations as fresh perspectives offered by locums led to different routes of treatment or management, and could alter organisational cultures or practices.

So that [locum] doctor, through that line of questioning and not having any sort of prior history … ordered the right tests and didn’t feel constrained in that practice about what tests that they could order. And someone subsequently … because when you get referred to hospital, the consultant said that that doctor was very much on the ball. And, of course, that’s a change to lifelong medication. And literally within a month of the medication kicking in, it transformed my life. (Focus group A, patient 1)

### Balance and stability

The balance between locum and permanent staff had implications for quality and safety, organisational leadership, long-term planning and governance. Locums were often employed to deliver immediate services and consequently were less likely to be involved in team and organisational development. Locums recognised that having ‘an NHS run by locums’ was detrimental to quality and safety, and some avoided organisations that were locum dependent for this reason. Well functioning established teams were regarded as better able to incorporate a small number of locums without being significantly impacted.

Locum work, my view on it is they’re there to fill a gap. They shouldn’t be relied upon to deliver a service Monday to Friday, day in, day out, week in, week out. And unfortunately my trust see it as that, though, that’s my worry that they feel they’re not just plugging a gap, they’re almost as a workforce … (Interview 84, lead GP, primary and secondary care)

Departments that were disproportionately locum dependent were often perceived to lack clinical leadership and direction. An absence of consistent medical leadership meant that quality improvement was slower or less likely to happen, and trusting relationships between staff were harder to establish.

If you get a department that is disproportionately locum dependent, then it stagnates, it doesn't progress. Things like implementation of new NICE guidance, for example, that sort of thing tends not to happen or happen less well, less quickly. (Interview 30, responsible officer, secondary care)

### Discrimination and exclusion

Most locums described negative behaviours and attitudes from staff and some patients, which impacted their involvement, inclusion and experiences in organisations. Negative attitudes and behaviours towards locums could affect turnover, locum well-being, team dynamics and potentially patient safety. Perceived disparities between pay, workload, competence and organisational and team commitment between locums and permanent staff could be sources of resentment and influenced how locums were treated and viewed. This compromised staff communication and reduced the sharing of important patient information.

I guess like any temporary post really, you struggle to invest in them, don't necessarily see them as being part of the team. Not very positive about them, particularly junior staff, particularly in the acute trusts. We'd have locums refusing to come back because of the treatment of the midwives. (Interview 86, clinical lead, secondary care)

Negative perceptions of competency and safety meant that locums were often stigmatised, marginalised and excluded. The identity of locum intersected and overlapped with other identities and was described as ‘layering up’ with ethnicity and gender to further exacerbate discrimination.

Oh, doctors coming over from Germany. There was one locum … that administered a dose of something and the patient died, and then there’s this whole layer of extra negativity attached to locum doctors in general because of what one doctor did, and that doctor happened to be someone from a different ethnicity … As a UK born and qualified doctor I can see that those overseas get it but I can also see that I have experienced that as well. So yeah, it can layer up with the whole locum thing. (Interview 59, locum GP, primary care)

A sense of othering and being seen as less was particularly evident during the COVID pandemic when resources were limited. Some locums described how they were not afforded the same protections as permanent members of staff and were sometimes expected to take on riskier work.

I’ve worked in another practice where, because they live on locums and they live on ad hoc locums, you’re a piece of dirt under the shoe. You don’t get gloves, you didn’t have aprons, you didn’t have a face visor, you didn’t have safety specs, you have to ask for a mask. Not only are you not treated as a service provider, you’re not treated as a colleague, someone with knowledge. (Interview 44, locum GP)

### Defensive practice by locums

Locums recognised that they were likely to be scapegoated if things went wrong, and some locums described being more likely to practice defensively. Defensive practice has been defined as deviation from standard practice to avoid litigation, complaints or criticism.^46^ Participants reported instances of defensive practice which involved providing services (eg, tests, referrals) or avoiding high-risk decisions, usually to reduce the risk of adverse outcomes such as patient complaints or potential termination of contract at short notice. Locums described practicing defensively because they were attempting to practice as safely as possible in complex unfamiliar environments where they were professionally isolated and perceived negatively. Permanent members of staff could perceive that locums practiced defensively because they lacked confidence in their abilities. The diversion of resources away from more clinically relevant activities placed additional burden on teams, who were already facing significant workload challenges.

Being risk averse and practising defensive medicine usually means more tests, more referrals, whereas holding risk tends to be disadvantageous for you as a locum because what’s the benefit to you of not doing that. You’re benefiting the system by rationing resource, the patient won’t thank you. (Interview 35, locum GP)

Locums described avoiding making decisions when risks to employment or medical licenses were perceived as high. Locums felt they were more vulnerable to criticisms of their clinical competence and disempowered to make decisions. Others felt that some locums were simply avoiding work and evaded responsibility for patients by pushing work onto others or into the future.

You don’t interfere, very simple. Over time locums have learned that if you interfere, if you participate in the team, you participate in patient care, [and this] is when you get into trouble … Well most of the locums that I know will just say, okay, there’s already somebody else who’s made a decision, it’s not my job to make a decision, I just follow through. If things go wrong, call the senior person and be done with it, that’s the end of my role. Actually doing something to protect a patient is not important for a locum because the risk is too high. (Interview 55, locum, secondary care)

### Locums fall outside clinical governance arrangements

Governance practices in relation to locums varied widely and were not generally regarded as being as robust in comparison to permanently employed doctors. Responsibility for involving locum doctors in performance feedback, supervision, educational opportunities, appraisal and quality improvement was unclear. While some organisations included locums in their governance activities, others regarded locum work as transactional; where the locum was there to provide a finite service and the organisation assumed no responsibilities for their performance, development or oversight. There were concerns that governance structures were modelled on and designed for permanently employed doctors and did not work for locums. When deficits in performance were undetected or unaddressed, doctor performance and patient safety could be jeopardised.

I think it’s a remote world. It’s like a cloud, you know, it’s like the cloud. We talk about the cloud when it comes to storing information. And I think locum world is a bit like that … And I don’t know the doctors anywhere like as much as I did when I was an RO in the NHS, I knew them all personally. If I used to have a problem, I used to get them in my office there and then, chat it all through, sort it. Can’t do that in locum world, it might take me four days to get hold of the doctor, some of them won’t respond immediately … They don’t know me and I don’t know them. (Interview 51, responsible officer, locum agency)

The absence of typical recruitment processes (involving meeting a doctor, carrying out an interview and following up on references) meant that healthcare organisations were reliant on partial information from locum agencies, which made it difficult to determine competency, scope of practice and suitability for a role. However, staff shortages and a requirement to meet safe staffing ratios meant that organisational leaders had little recourse of action if they were unsure about a doctor’s capability, which caused anxiety and frustration. This suggests that the provision of healthcare superseded ensuring safety standards and necessitated accepting one of two objectionable alternatives; accepting gaps in staffing that may jeopardise patient safety or accepting unknown doctors; each of which may compromise patient safety.

If a locum turns up and I have serious doubts about their ability to do the job to the required standard, I don’t have any recourse … And therefore I’m in a position where either I accept this locum or I don’t. There’s not much in the way of middle ground. Not accepting them is a really unpalatable choice because if I say look, I’m sorry, I don’t think you’re up to this, I think you should go home, that leaves me with a gap. (Interview 30, consultant and responsible officer, secondary care)

Similar governance and information sharing problems were described by locum agencies and NHS organisations; both described difficulties in gathering and sharing feedback. When concerns were raised, participants were often uncertain as to what happened to the information they provided and whether it was shared or acted on. Locums often did not get to hear about concerns raised about them, meaning learning opportunities were missed.

It would give you more confidence if you heard back. And sometimes I'll pick up the phone and you try to do the best you can to make sure this information gets passed on. But I just have this nagging doubt that I'm not always convinced it does. (Interview 30, responsible officer, secondary care)

There was also a perception from some locum agency responsible officers that while most locum doctors were excellent, there were some locums who were isolated and in need of organisational and professional support.

You have to accept that whilst within the agency world, 80 per cent of the doctors we place are excellent, and have no problems, and do a great job, perhaps 20 per cent are those that have shaken down to that 20 per cent in the agency world, because they’ve not succeeded in the NHS, they’ve not got a substantive place, they are lost souls. And they are less able to cope with the vicissitudes of busy clinical life and professional life within a large organisation such as the NHS. (Interview 47, responsible officer, locum agency)

## Discussion

Our findings provide some profound and concerning insights for patient safety and quality of care. The ways in which locums were recruited, inducted, deployed and integrated, and supported by organisations undoubtedly affected quality and safety. Our findings indicate that regardless of their level of experience, it was unlikely that locum doctors would be able to function optimally in unfamiliar environments; and organisations who had poor supportive infrastructure and governance mechanisms for locums were less likely to deliver high-quality safe services.

Locums were often regarded as organisational outsiders—positioned at the periphery of the team and the organisation. The implications of transience and peripheral participation were weaker relationships with organisations, teams, peers and patients, leading some to suggest locum working is better suited to experienced doctors.^47^ Consistent with previous research,^48^ frequent variation in process, systems and equipment, combined with disruption in relationships and a lack of mutual awareness of team skills and competencies, decreased collective competence, placed additional burden on the wider healthcare team and reduced patient safety. As others have found in research on safe staffing and nursing,^49^ temporary staff are not effective substitutes for staff who regularly work in the organisation. Safe medical staffing is not just achieved by filling rota gaps, but also team composition and doctors’ familiarity with the team and organisation must be taken into account. Regulatory agencies should consider locum usage in their inspections and perhaps be particularly concerned when organisations have ‘services run on locums’.

Our research found, as others have,^18^ that organisations and doctors sometimes struggled to meet their governance obligations and that governance activities differed based on contractual status and organisational policies and norms, with systems being less robust for locums. This research has highlighted that much still needs to be done to develop governance systems that promote and protect the interests of patients and create an environment which supports locum doctors in meeting their professional obligations.

More positively, locum doctors are a potentially valuable source of information about safety concerns, faulty systems or poor conduct.^50^ Locums move between organisations, have broad systems knowledge and are perhaps better placed to identify some quality and safety issues than permanent doctors. However, findings indicate opportunities for shared learning were often missed. Locums recognised their precarity and vulnerability when offering second opinions, sharing improvement ideas or voicing safety concerns; meaning opinions were not always offered and concerns were not always raised. Failure to voice concerns is a persistent problem in healthcare,^51^ and locums may be even less inclined to offer potentially valuable information about safety concerns because of their perceptions of unsupportive organisational climates.

Our findings shed light on how temporary doctors fit into the enduring debate^37^ around how responsibility between organisational systems and individual professionals is distributed. Locums appear to represent a subsection of the medical profession for whom the wider paradigm shift from a focus on individual blame to a systems approach^52^ appeared not to have been made. Locums were often not regarded as a part of the organisation, and therefore the system, and not afforded the same protections as permanent staff when things went wrong. Blaming locums when things go wrong and punishing or sanctioning individuals who make errors in contexts that were not designed to incorporate temporary workers may divert attention from understanding inadequately designed, poorly functioning systems, or indeed the individual practice of other doctors. While we should take into account systemic factors that impede locums from performing safely, we should expect high standards of healthcare professionals, be cognisant of individual agency and recognise the distinction between blaming someone and holding them responsible.^53^

### Strengths and limitations

This large qualitative study explores locum working and quality and safety in an under-researched, yet growing area of the medical workforce. However, sites were all based in England, which means caution should be taken when extrapolating findings. Similar research in other countries and contexts to understand more about locum doctor working and quality and safety is therefore important. It is possible that our sample may have been skewed towards locums, healthcare professionals and patients who had more negative perceptions and experiences, although accounts resonate with previous research^30^ and patient perspectives were generally positive. Our data were collected during the COVID pandemic, which may have affected findings as there was a reduction in locum working during that time^10 11^; it also meant we were unable to carry out observations, which would have strengthened our findings and mitigated some of the inherent limitations of interviews, such as recall bias. We used both one-to-one interviews and focus groups in data collection. Although flexibility in data collection meant that participants had the option to take part in an interview or a focus group, these methods are used for different reasons and produce different data. There may have been differences in what participants disclosed depending on the method

## Conclusion

Our findings show that the way in which doctors who worked on a temporary basis were integrated into organisations posed some significant challenges and opportunities for patient safety and quality of care, and that both organisations and locums had a part to play in improvement. Doctors working as locums are a heterogeneous group with differing backgrounds, experiences, skills and capabilities that likely reflect the variability seen in the wider population of doctors. Locums are working in the same pressured and imperfect systems as other health workers; it is vital that systemic problems are not mistaken for problems about individuals and important to recognise that a locum is not a type of doctor but a way of working. Our findings are a call to action for organisations to take stock of how they engage, support and work with locums, and asks both locums and organisations to reflect on whether their practices support a collective approach to patient safety and quality of care.

10.1136/bmjqs-2023-016699.supp4 Supplementary data

## Data Availability

No data are available.
