# Supplementary material for: Locum doctor working and quality and safety: a qualitative study in English primary and secondary care
Source: BMJ Qual Saf. 2024 Apr 16;33(6):354–62. doi: 10.1136/bmjqs-2023-016699 (PMC11103325; doi:10.1136/bmjqs-2023-016699)
Supplement: Supplementary data [file bmjqs-2023-016699supp001.pdf]

FUNDED BY

The use of locum doctors in the NHS: understanding and improving the safety and quality of care

Locum Doctor Interview Schedule

Thank you for agreeing to take part in this project. I'd like to confirm that you have read the participant information sheet and check whether you have any questions about the study? This interview/focus group should last approximately one hour. We'd like to remind you that the information collected from interviews and focus groups will be kept strictly confidential, and your responses will be pseudonymised, so you will not be identifiable in any published data. Any discussions that take place during the study are confidential. However, if you were to tell us something that could put someone at risk of harm, or reveal unsafe practice that has not been reported through the usual procedures the researcher might be professionally obliged to report the incident through the normal risk management procedures. Information that indicates harm to patients or professional misconduct will be disclosed by the research team as part of a safeguarding process, in accordance with established good research practice and with the University of Manchester's own policy on whistleblowing and public interest disclosure. If this happens, the interview will be stopped and we will discuss with you what we intend to do.

Narrative question

Could you spend 5-10 minutes telling me about your career?

What are your plans for the future?

Prompt: can you tell me more about your decision to become a locum doctor?

Finding work and induction

How do you find work? e.g. do you work with an agency? Word of mouth? How do you choose the agencies you work for?

Tell me about the places you work in? How do you choose them?

Are you typically offered induction? Are you typically offered a paid induction?

Tell me about your experiences of induction e.g. are you provided with details of where things are, who people are, login details and/or more complete induction processes

What does a good induction look like?

What should be included in induction to make it safer/easier for you to work?

IRAS title: The use of locum doctors in the NHS
IRAS ID: 278888
Version 1 13/01/2021

Experiences of locum working

What would you say the key differences were between working permanently and working as a locum? Prompts: What are the advantages and disadvantages of locum work for you? What are the advantages and disadvantages of locum work for patients?

What have been your pre-covid and during the pandemic experiences of being a locum?

Do patients know you are a locum? Do you tell them?

How do patients usually respond to you as a locum?

How do staff respond?

Are you usually included in MDT activities, meetings and/or educational activities?

What types of staff training if any are you offered? E.g. statutory/mandatory e.g. information governance; general professional basics e.g. safeguarding and basic life support; general CPD opportunities

Who do you revalidate through? Do you feel well supported in this? Has this been impacted by the pandemic?

For a provider where you have not worked before do you undertake any preparatory work?

How are you supported in your clinical work? E.g. peer clinical advice and/or navigating the local system
Prompt: who do you usually go to if you need support?

What has happened if there was a clinical problem with your work?

What has happened if there was a general problem with your work?

Do you get to hear about complements and complaints?

What happens when you reach the end of placement? Do you get feedback?

Critical incident question

The overall aim of this research is to provide evidence on the quality and safety of medical locum practice and the implications of medical locum working for health service organisation and delivery. Can you describe a time, *whether it be positive or negative*, when working as a locum, or how your work was organised by the organisation you worked for, had implications for the quality and safety of care?

IRAS title: The use of locum doctors in the NHS
IRAS ID: 278888
Version 1 13/01/2021

FUNDED BY NIHR | National Institute for Health Research

Probes for critical incident question
What happened next?
Who was involved?
What did the organisation do?
What was the outcome?
How did that make you feel?
Why did this situation happen in this way?
What could have made the action more effective?

Locum working and the pandemic

What has been the impact of the pandemic on your work as a locum?

Initiatives

Are you aware of any policies or initiatives that are being used or developed to improve how locums are used by organisations?

What would you like to see included in any initiatives?

Anything else?

Is there anything else you'd like to add or anything that we haven't covered?

IRAS title: The use of locum doctors in the NHS
IRAS ID: 278888
Version 1 13/01/2021
