# Supplementary material for: Locum doctor working and quality and safety: a qualitative study in English primary and secondary care
Source: BMJ Qual Saf. 2024 Apr 16;33(6):354–62. doi: 10.1136/bmjqs-2023-016699 (PMC11103325; doi:10.1136/bmjqs-2023-016699)
Supplement: Supplementary data [file bmjqs-2023-016699supp002.pdf]

MANCHESTER 1824
The University of Manchester

FUNDED BY NIHR | National Institute for Health Research

The use of locum doctors in the NHS: understanding and improving the safety and quality of care

Focus Group Patient and Carer Schedule

Note to facilitators: Please refer back to these notes just before the group is due to meet to refresh your memory.

One member of the project PPI forum will lead the group and ask the questions, while a member of the research team will support the interviewer. The research team will take responsibility for the recruitment of participants, issuing participants with participant information sheets and consent forms, taking consent from focus group participants, handling the recording equipment, make field notes and transferring sound files.

Remember to take a note of the date and case study site that the focus group is related to and record on the audio.

It is important to remember that you are seeking to explore the viewpoint of the group. You should try to get everyone involved in the discussion; however, this does not mean that everyone must have the same view. You need to explore both majority and minority views.

Before the group assembles

The research team will ensure participants have received and completed the relevant paperwork before the focus group meets, e.g. participant information sheets and consent forms, P20 forms so they can claim payment. They will test the recording equipment to make sure it is working and that the sound is recording at an acceptable level.

Preparing to start the session

If the focus group is taking place by a platform such as Zoom or MS Teams, to encourage discussion, ask all participants to keep their microphones and cameras on wherever possible. To facilitate discussion virtually, focus groups will be kept to a maximum of four participants.

Once people are settled, check with the group whether they all know each other. If not, start by going round the group and getting everyone to introduce themselves.

Make sure that everyone is comfortable before you start and that everyone can see and hear each other. Read out the statement on confidentiality:

Opinions expressed will be treated in confidence among project staff for the purpose of understanding patient and carer experiences and views of locum doctors. All responses will remain anonymous. Please don't refer to any individual patients or doctors by name or name a specific hospital or practices. Also, please don't describe any serious unsafe practices that led to serious harm that haven't already been dealt with through the proper channels as we will be obliged to report it.

IRAS title: The use of locum doctors in the NHS
IRAS ID: 278888
Version 1 17/12/2020

FUNDED BY NIHR | National Institute for Health Research

Check that there are no objections to the use of the audio recorder; then Jane or Gemma will switch it on.

Introduction to the session

Start off by reiterating the purpose of the focus group:

I'm very grateful to you all for sparing time to talk about your views and experiences of locum doctors and how locum working might impact care. The purpose of this focus group is to better understand patients' and carers experiences of locum doctors and their views of locum working. This understanding will help to find ways to improve the working arrangements for locum doctors and the quality and safety of patient care that they provide. I would like to concentrate on discussing what you think about locums before going on to discuss any experiences you might have had. There are no right or wrong opinions, this discussion is confidential and we would like you to feel comfortable saying what you really think and how you really feel.

Perceptions of locum working

What comes to mind when you think about locum doctors?

Prompt: What do you think a locum doctor is? (i.e. how would you define one).

Accessing care

Do the group think patients and carers generally know when they are seeing a locum?

Prompt: How did you know they were a locum?

Do you think you should be told when the doctor is a locum? If so, why?

Have you ever made another appointment with your regular doctor after seeing a locum? If so, why/why not?

Have you ever refused an appointment because it was with a locum? If so, why?

Have you ever not gone to the doctor because your regular doctor wasn't available? If so, why/why not?

Are there particular circumstances when you would prefer to see your regular doctor or permanently employed doctor rather than a locum? Which circumstances would you be happy to see a locum, and when would you rather see your regular doctor?

Critical incident question: experiences of locum doctors

The overall aim of this research is to better understand the quality and safety of locum practice and how locum working might affect care. Can you describe a time, whether it be positive or negative, when care was provided by a locum?

IRAS title: The use of locum doctors in the NHS
IRAS ID: 278888
Version 1 17/12/2020

Do you think there are any differences in how care is provided when permanent staff are employed in comparison to when locums are employed? Can you describe any differences and what these differences have meant for you or the person you were caring for?

Prompt: If there was a problem, what happened next? How was it dealt with?

Challenges and opportunities

Why do you think some organisations are more reliant on locums than others?

What challenges do you think a locum doctor might face?

What would a good consultation with a locum look like?

Because they might not know you, are there any key questions the locum should ask?

How do you think locums should be supported to provide safer care?

What do you think should change to make care safer for patients when locums are delivering care?

Anything else

Has taking part in this focus group changed how think about locum doctors/ changed how you might make appointments in the future?

If there anything we haven't covered that you would like to talk about?

Summarise the discussion and check in with participants if there are any key issues that study team should focus on.

Ending the session

Finally, thank participants for their time and reiterate that the discussion is confidential.

Submit the recording to the transcription service.

IRAS title: The use of locum doctors in the NHS
IRAS ID: 278888
Version 1 17/12/2020
