# Supplementary material for: Locum doctor working and quality and safety: a qualitative study in English primary and secondary care
Source: BMJ Qual Saf. 2024 Apr 16;33(6):354–62. doi: 10.1136/bmjqs-2023-016699 (PMC11103325; doi:10.1136/bmjqs-2023-016699)
Supplement: Supplementary data [file bmjqs-2023-016699supp003.pdf]

The use of locum doctors in the NHS: understanding and improving the safety and quality of care

Professionals Interview Schedule

Thank you for agreeing to take part in this project. I'd like to confirm that you have read the participant information sheet and check whether you have any questions about the study? This interview should last approximately one hour. We'd like to remind you that the information collected from interviews will be kept strictly confidential, and your responses will be pseudonymised, so you will not be identifiable in any published data. Any discussions that take place during the study are confidential. However, if you were to tell us something that could put someone at risk of harm, or reveal unsafe practice that has not been reported through the usual procedures the researcher might be professionally obliged to report the incident through the normal risk management procedures. Information that indicates harm to patients or professional misconduct will be disclosed by the research team as part of a safeguarding process, in accordance with established good research practice and with the University of Manchester's own policy on whistleblowing and public interest disclosure. If this happens, the interview will be stopped and we will discuss with you what we intend to do.

How does your job relate to locum working?

Tell me about what you do and how your job relates to locums? E.g. do you have hiring responsibilities, governance responsibilities, do you work alongside locums?

[Depending on the nature of the involvement with locum doctors] Can you describe the process of recruitment/induction/governance/integration with the team?

Perceptions of locum working

When you think about locums what comes to mind?

Why do you think doctors choose to work as locums?

Would you say there were different 'types' of locum?

Have you ever worked as a locum? If so, why?

Would you ever work as a locum? Why? Why not?

How do staff respond to locums?

Why are locums needed where you work?

Why are locums needed in your organisation?

Where are they most likely to work?

Does the need for locums differ across specialities? Why?

What types of work do they usually do?

IRAS title: The use of locum doctors in the NHS
IRAS ID: 278888
Version 1 15/12/2020

Patients and locums

How do patients respond to locum doctors?

Do patients know when they're seeing a locum?

Do you think patients should know or need to know?

Do locums typically find out if patients complement them or complain about them?

Critical incident question

The overall aim of this research is to provide evidence on the quality and safety of medical locum practice and the implications of medical locum working for health service organisation and delivery. Can you describe a time, *whether it be positive or negative*, when locum working, or how a locum was engaged by your organisation, has had implications for the quality and safety of care?

Do you think there are any differences in how services are delivered when permanent staff are employed in comparison to when locums are employed? Can you describe any differences and the implications?

What happens when things go wrong or right?

If there is a problem with a locum, what happens next?

Prompt: What happens if there is a complaint or an SUI involving a locum? What do you do in this situation?

Would you say there are any typical issues when it comes to locum working? What are they?

Does the locum typically find out if they were involved in an SUI?

Prompts: Who deals with this?

What benefits do locums bring to your organisation?

How do you capture their knowledge and share it with others?

Governance and support for locums

How does your organisation support locums?

What sort of support do you think locums should have?

Are locums included in CPD where you work?

Do locums attend MDT meetings and events?

IRAS title: The use of locum doctors in the NHS
IRAS ID: 278888
Version 1 15/12/2020

Impact of the pandemic

How has the pandemic impacted on locum working where you work?

Initiatives

Are you aware of any policies or initiatives that are being used or developed to improve how locums are used by organisations?

What would you like to see included in any initiatives?

Anything else?

Is there anything else you'd like to add or anything that we haven't covered?

Thank you for taking part

IRAS title: The use of locum doctors in the NHS
IRAS ID: 278888
Version 1 15/12/2020
