# Supplementary material for: Locum doctor working and quality and safety: a qualitative study in English primary and secondary care
Source: BMJ Qual Saf. 2024 Apr 16;33(6):354–62. doi: 10.1136/bmjqs-2023-016699 (PMC11103325; doi:10.1136/bmjqs-2023-016699)
Supplement: Supplementary data [file bmjqs-2023-016699supp004.pdf]

Consolidated criteria for reporting qualitative studies (COREQ): 32-item checklist

No. Item	Guide questions/description	Reported on Page #
**Domain 1: Research team and reflexivity**		
Personal Characteristics		
1. Inter viewer/facilitator	Which author/s conducted the interview or focus group?	#4
2. Credentials	What were the researcher's credentials?	#1 Authors details and credentials
3. Occupation	What was their occupation at the time of the study?	#1 Authors details and credentials
4. Gender	Was the researcher male or female?	#1 Authors details and credentials
5. Experience and training	What experience or training did the researcher have?	#4
Relationship with participants		
6. Relationship established	Was a relationship established prior to study commencement?	No.
7. Participant knowledge of the interviewer	What did the participants know about the researcher? e.g. personal goals, reasons for doing the research	n/a
8. Interviewer characteristics	What characteristics were reported about the inter viewer/facilitator? e.g. Bias, assumptions, reasons and interests in the research topic.	#5

**Domain 2: study design**		
Theoretical framework		
9. Methodological orientation and Theory	What methodological orientation was stated to underpin the study? e.g. grounded theory, discourse analysis, ethnography, phenomenology, content analysis.	#5
Participant selection		
10. Sampling	How were participants selected? e.g. purposive, convenience, consecutive, snowball	#3
11. Method of approach	How were participants approached? e.g. face-to-face, telephone, mail, email	#3
12. Sample size	How many participants were in the study?	#4
13. Non-participation	How many people refused to participate or dropped out? Reasons?	n/a
Setting		
14. Setting of data collection	Where was the data collected? e.g. home, clinic, workplace	#5
15. Presence of non-participants	Was anyone else present besides the participants and researchers?<br><br>No	n/a
16. Description of sample	What are the important characteristics of the sample? e.g. demographic data, date	#4<br>Tables 1 and 2
Data collection		
17. Interview guide	Were questions, prompts, guides provided by the authors? Was it pilot tested?	#3
18. Repeat interviews	Were repeat inter views carried out? If yes, how many?	**n/a**
19. Audio/visual recording	Did the research use audio or visual recording to collect the data?	#4

20. Field notes	Were field notes made during and/or after the interview or focus group?	#5
21. Duration	What was the duration of the inter views or focus group?	#5
22. Data saturation	Was data saturation discussed?<br><br>No. The notion of data saturation is not consistent with the values and assumptions of reflexive thematic analysis.	n/a
23. Transcripts returned	Were transcripts returned to participants for comment and/or correction?<br><br>No.	n/a
**Domain 3: analysis and findings**		
Data analysis		
24. Number of data coders	How many data coders coded the data?	#5
25. Description of the coding tree	Did authors provide a description of the coding tree?	#5
26. Derivation of themes	Were themes identified in advance or derived from the data?	#5
27. Software	What software, if applicable, was used to manage the data?	#5
28. Participant checking	Did participants provide feedback on the findings?	No
Reporting		
29. Quotations presented	Were participant quotations presented to illustrate the themes/findings? Was each quotation identified? e.g. participant number	#6-9
30. Data and findings consistent	Was there consistency between the data presented and the findings?	#6-9
31. Clarity of major themes	Were major themes clearly presented in the findings?	#6-9
32. Clarity of minor themes	Is there a description of diverse cases or discussion of minor themes?	#6-9
